# Sex Specific Association of Apolipoprotein E Alleles with Autopsy Measures of Arteriolosclerosis and Atherosclerosis among Older Adults

**DOI:** 10.1002/alz.091485

**Published:** 2025-01-03

**Authors:** Melisa Lara Gomez, Gary Beecham, Thomas J. Montine, Michael L. Cuccaro, Arthur W. Toga, C Dirk Keene, Paul K. Crane, Shubhabrata Mukherjee, Matthew S. Panizzon, Sarah A Biber, Walter A. Kukull, David A. Bennett, Lisa L. Barnes, Julie A. Schneider, Philip L. De Jager, Vilas Menon, Timothy J. Hohman, Logan C. Dumitrescu

**Affiliations:** ^1^ Vanderbilt Memory & Alzheimer’s Center, Vanderbilt University Medical Center, Nashville, TN USA; ^2^ Vanderbilt Genetics Institute, Vanderbilt University Medical Center, Nashville, TN USA; ^3^ Dr. John T. Macdonald Foundation Department of Human Genetics, University of Miami Miller School of Medicine, Miami, FL USA; ^4^ Department of Pathology, Stanford University School of Medicine, Stanford, CA USA; ^5^ John P. Hussman Institute for Human Genomics, University of Miami Miller School of Medicine, Miami, FL USA; ^6^ Laboratory of Neuro Imaging, USC Stevens Neuroimaging and Informatics Institute, Keck School of Medicine, University of Southern California, Los Angeles, CA USA; ^7^ Department of Laboratory Medicine and Pathology, University of Washington, Seattle, WA USA; ^8^ Department of Medicine, University of Washington, Seattle, WA USA; ^9^ Center for Behavior Genetics of Aging, University of California, San Diego, La Jolla, CA USA; ^10^ University of Washington, Seattle, WA USA; ^11^ Department of Epidemiology, School of Public Health, University of Washington, Seattle, WA USA; ^12^ Rush University, Chicago, IL USA; ^13^ Rush University Medical Center, Chicago, IL USA; ^14^ Department of Neurology and Pathology, Rush University Medical Center, Chicago, IL USA; ^15^ Department of Neurology, Center for Translational and Computational Neuroimmunology, Columbia University Medical Center, New York, NY USA; ^16^ Center for Translational & Computational Neuroimmunology, Department of Neurology, Columbia University Irving Medical Center, New York, NY USA; ^17^ Vanderbilt Memory & Alzheimer’s Center and Vanderbilt Genetics Institute, Vanderbilt University Medical Center, Nashville, TN USA; ^18^ Vanderbilt Memory and Alzheimer’s Center, Vanderbilt University Medical Center, Nashville, TN USA

## Abstract

**Background:**

Cerebrovascular disease (CVD) is a major cause of mortality in females, while two‐thirds of Alzheimer’s disease (AD) patients are female. AD and CVD share many genetic risk factors, one of them being apolipoprotein E (*APOE*) genotype. Sex differences in *APOE* and AD are well‐established; it is unclear if associations between *APOE* and CVD are sex‐specific.

**Method:**

Autopsy data (females = 3,464; males = 3,130) from three AD cohorts included measures of arteriolosclerosis and atherosclerosis (levels for each: none [0], mild [1], moderate [2], and severe [3]) harmonized as part of the AD Sequencing Project Phenotype Harmonization Consortium. Non‐Hispanic White (NHW) individuals >60 years at age of death were included (average age at death = 85±9; 58% AD dementia). Linear analyses assessed sex‐stratified and APOE*sex interactions on CVD outcomes covarying for age at death. *APOE*‐ε4 and *APOE*‐ε2 were modeled additively and dominantly, respectively.

**Result:**

Females were older on average and included more *APOE*‐ε2 and less *APOE*‐ε4 carriers when compared to males. Among males and females, *APOE*‐ε2 was not related to arteriolosclerosis (p = 0.90) or atherosclerosis (p = 0.30), while *APOE*‐ε4 related to higher arteriolosclerosis (β = 0.07 p<0.001) and atherosclerosis (β = 0.04, p = 0.03). There was a significant *APOE*‐ε2*sex interaction on arteriolosclerosis (p = 0.02) whereby the ε2 allele was associated with less arteriolosclerosis among females (β = ‐0.05, p = 0.27) and more arteriolosclerosis among males (β = 0.08, p = 0.13), although neither stratified effect reached statistical significance. Similarly, the ε4 allele was associated with more arteriolosclerosis among females (β = 0.11, p<0.001), but not among males (β = 0.04, p = 0.16), although the *APOE*‐ε4*sex interaction did not reach statistical significance (interaction‐p = 0.052). The ε4 allele was associated with more atherosclerosis among females (β = 0.07, p = 0.01), but not males (β = 0.01, p = 0.56), but again the *APOE*‐ε4*sex interaction did not reach statistical significance (p = 0.28).

**Conclusion:**

We provide evidence that the ε4 allele is related to higher levels of arteriolosclerosis and atherosclerosis, while providing modest evidence of a sex difference in the association between the ε2 allele and arteriolosclerosis. Future work will seek to better characterize these sex‐specific effects within the context of other neurodegenerative pathologies in the aging brain, and more fully characterize sex‐specific clinical consequences of *APOE* and CVD.

